# Website Quality Indicators for Consumers

**DOI:** 10.2196/jmir.7.5.e55

**Published:** 2005-11-15

**Authors:** Kathleen M Griffiths, Helen Christensen

**Affiliations:** ^1^Depression & Anxiety Consumer Research UnitCentre for Mental Health ResearchThe Australian National UniversityCanberraAustralia; ^2^Centre for Mental Health ResearchThe Australian National UniversityCanberraAustralia

**Keywords:** Depressive disorder, medical informatics, consumer participation, evaluation studies

## Abstract

**Background:**

The rating tool DISCERN was designed for use by consumers without content expertise to evaluate the quality of health information. There is some evidence that DISCERN may be a valid indicator of evidence-based website quality when applied by health professionals. However, it is not known if the tool is a valid measure of evidence-based quality when used by consumers. Since it is a lengthy instrument requiring training in its use, DISCERN may prove impractical for use by the typical consumer. It is therefore important to explore the validity of other simpler potential indicators of site quality such as Google PageRank.

**Objective:**

This study aimed to determine (1) whether the instrument DISCERN is a valid indicator of evidence-based Web content quality for consumers without specific mental health training, and (2) whether Google PageRank is an indicator of website content quality as measured by an evidence-based gold standard.

**Methods:**

This was a cross-sectional survey of depression websites using consumer and health professional raters. The main outcome measures were (1) site characteristics, (2) evidence-based quality of content as measured by evidence-based depression guidelines, (3) DISCERN scores, (4) Google PageRank, and (5) user satisfaction.

**Results:**

There was a significant association between evidence-based quality ratings and average DISCERN ratings both for consumers (*r* = 0.62, *P* = .001) and health professionals (*r* = 0.80, *P* < .001). Consumer and health professional DISCERN ratings were significantly correlated (*r* = 0.77, *P* < .001). The evidence-based quality score correlated with Google PageRank (*r* = 0.59, *P* = .002). However, the correlation between DISCERN scores and user satisfaction was higher than the correlation between Google PageRank and user satisfaction.

**Conclusions:**

DISCERN has potential as an indicator of content quality when used either by experts or by consumers. Google PageRank shows some promise as an automatic indicator of quality.

## Introduction

There has been widespread concern about the quality of Web-based health information designed for consumers [1]. In response to this, a number of initiatives have been developed to assist consumers in locating quality health information on the Web. These include the use of quality labels based on compliance with codes of conduct (eg, HON code), portals that provide a gateway to websites of “high quality” (eg, OMNI), and rating tools designed for consumer use [2].

One rating tool that shows particular promise is DISCERN, an instrument designed for use by consumers and providers “to judge the quality of written information about treatment choices” [3, p. 106]. This tool is widely recommended and used by authoritative sources for the evaluation of websites. However, it has not yet been convincingly established that DISCERN, particularly when used by consumers, is a valid indicator of quality when compared against an evidence-based gold standard.

Three studies have investigated the relationship between the DISCERN ratings of experts and “scientific” quality [4-6]. Two of the studies reported a significant association between DISCERN and scientific accuracy [4,5], but the authors of the third study found “no clear relationship between methodological (DISCERN) and medical-scientific quality” [6]. Unfortunately, except for the Griffiths and Christensen study [4], it is unclear if the standard against which the DISCERN ratings were compared was based on systematic reviews of the evidence. Moreover, in each study, ratings were made by health professionals. To date, to our knowledge, there has been no assessment of the validity of DISCERN as measured by an evidence-based gold standard when used by consumers without technical expertise.

Although the developers trialed DISCERN with self-help group users in a research context, it is a lengthy instrument, and it is not clear if individual consumers would use DISCERN in practice. Other simpler potential indicators of site quality include those based on the link structure of the World Wide Web. For example, Google PageRank is an automatically computed measure of the importance of a website based on the number and importance of Web pages linking to it. However, there is little evidence as to the validity of link structure as an indicator of quality.

The current study, therefore, sought to determine the following for depression information websites: (1) whether DISCERN is a valid indicator of evidence-based content quality for consumers without specific mental health training, and (2) whether Google PageRank is an indicator of content quality. Depression websites were selected because depression is a leading cause of disease burden [7], there is a high level of unmet need among people with depression [8], depression is one of the most common reasons consumers access health information on the Internet [9], and evidence-based guidelines for depression management are available.

## Methods

### Website Selection

Twenty-four depression websites with a Google PageRank were selected from the Depression Directory of the DMOZ Open Directory Project website (n = 127). Three sites for each Google PageRank score within the range 0 to 7 were randomly selected using the R Project statistical package [10] to ensure a range of sites were represented. Each of the selected sites was then captured (in April 2003) and electronically archived for assessment using purpose built software. External links from these sites were excluded.

### Site Assessment

Sites were rated online by four researchers/health professionals with expertise in depression and three consumers with a history of depression but no professional experience in mental health or research. Two of the health professionals (KG, HC) rated the site using an evidence-based gold standard. They also rated the characteristics of each website. The other two health professionals (AJ, RK) and the three consumers rated the sites using the DISCERN measures. All raters provided satisfaction measures for each site. Sites were presented in a different random order for each rater, and each rater was supplied with a pro forma rating sheet. The consumer raters were employed as casual research assistants during the study.

### Site Characteristics

Each site was rated on a range of attributes, including ownership structure, scope, editorial arrangement, and legal policies (Table 1).

### Evidence-Based Guideline Score

Evidence-based quality was assessed using the depression guidelines produced by the Centre for Evidence Based Mental Health (CEBMH) at Oxford [11]. The guideline score was the number of CEBMH items (maximum 20) correctly endorsed by the website [4]. In the current study, the correlation between evidence-based guideline scores for the two health professional raters was 0.94 (*P* < .001). An average guideline score was therefore computed for the two raters.

### DISCERN Scores

The DISCERN instrument comprises 15 items (each rated from 1 to 5) and an additional “overall quality” item (rated 1 to 5) [3,12]. Raters in the current study were informed that the DISCERN questionnaire was designed to assess the quality of information about medical treatments and that “In this study we are focusing on the quality of web sites related to the treatment of depression.” Each rater was provided with the DISCERN instrument, which includes hints for rating each item, and the DISCERN handbook, which contains detailed information about the scoring of DISCERN items. Items in DISCERN include questions about the reliability of the publication (eg, are information sources specified, is it clear where these information sources were produced, degree to which the discussion is balanced) and the quality of information on treatments (eg, description of the mechanism, benefits, risks of possible choices and inclusion of multiple treatment options).

Previous research has demonstrated acceptable inter-rater agreement on individual items of the instrument when used by expert health professionals and “fair” agreement among consumers [3]. The original version of the test used the overall quality score as the measure of quality. However, subsequently, a number of studies employing DISCERN have used a measure of quality based on a total DISCERN score derived by cumulating scores across the first 15 DISCERN items (minimum score = 15; maximum = 75) (eg, [4,5,13,14]). This measure shows acceptable inter-rater agreement (*r* = 0.88 [4], r = 0.82 [14]) and has been reported to correlate with the overall quality rating (*r* = 0.8 [14]). In the current study, the correlation between the total DISCERN score and the overall quality item score was 0.91 for consumers and 0.92 for experts. The DISCERN results reported in the primary analyses are therefore confined to the total DISCERN measure.

The correlation between the DISCERN ratings for the two health professionals was 0.86 (*P* < .001). Intercorrelations between DISCERN ratings for the three consumer raters were 0.78, 0.77, and 0.68 (*P* < .001). An average score was therefore computed for the health professionals and the consumers. The DISCERN ratings for the health professionals and consumers were significantly correlated (*r* = 0.77, *P* < .001). A paired *t* test demonstrated that mean DISCERN scores for the two types of rater did not differ significantly across the 24 websites (*t*_23_ = 0.64, *P* = .53).

### Satisfaction

Website satisfaction was measured using a series of 9 items developed for the purpose of the study. Items included questions about the target website’s perceived usefulness, relevance to people with depression, trustworthiness, author knowledge, esthetics, and whether the site could be easily understood, easily navigated, and would be recommended. A total satisfaction score was calculated by computing the total number of satisfaction items endorsed by the rater (minimum 0, maximum 9). The correlation between satisfaction ratings for the two evidence-based guideline health professional raters was 0.86 (*P* < .001) and for the two DISCERN health professional raters was 0.83. Intercorrelations between satisfaction measures for the three consumer raters were significant in two of the three cases (rater 1 vs 2: *r* = 0.60, *P* = .002; rater 2 vs 3: *r* = 0.58, *P* = .003; rater 1 vs 3: *r* = 0.26, *P* = .22). Therefore, although the satisfaction measure for the evidence-based guideline health professional raters was based on their average score, and an average score was also computed for the DISCERN health professional raters, the satisfaction measures for the three consumer raters were treated separately.

### Google ToolBar PageRank

Google PageRank is employed by the Google search engine as a measure of the “importance” of a Web page. These PageRank values can range from 0 to 10, with higher values indicating greater importance. PageRanks are based on an iterative algorithm developed by Google founders Brin and Page [15] that takes into account the number and importance of pages which link to a website. The importance of pages linking to a site is assessed according to the number and importance of sites linking to those pages. The PageRank score on the Google toolbar is a transformed function (conjectured to be logarithmic or distributional) of a raw Google PageRank score. The latter are very small positive numbers which sum to 1.0 over the entire Web and are known to be power-law distributed [16]. Google PageRank differs from the ranking order in Google search results in that PageRank is query independent, whereas the ranking order in Google search results takes into account many other variables, such as frequency of occurrence of search terms on a page, anchor text used to link to sites, and a large number of other tuning variables not disclosed by the company, as well as PageRank.

The Google PageRank for each site was obtained by downloading the Google toolbar and recording the integer number attached to the toolbar. The lowest and highest identified page ranks in the DMOZ depression directory were 0 and 7, respectively.

### Analyses

Intercorrelations between evidence-based scores, DISCERN, and overall satisfaction were computed using Pearson r tests. (Note that when these analyses were recomputed using non-parametric Spearman rho tests, similar patterns of results were observed.) Site quality was assessed as a function of site characteristic using independent *t* tests (with Levene’s correction in the case of unequal variances). Differences between evidence-based scores as a function of individual satisfaction items were analyzed separately using independent *t* tests except that no analysis was performed for items for which the sample sizes in a cell were very small (less than 6 sites). Multiple independent *t* tests were also used in analyzing the effects of site characteristics and for individual satisfaction items because the data were not amenable to an overall multivariate analysis such as a multiple regression or a MANOVA followed by contrasts corrected for multiple comparisons. For example, there were insufficient websites given the number of independent predictors to apply multiple regression to the data. The probability values cited in the results tables and text therefore refer to error rate per comparison. Given that a large number of comparisons were conducted in this study, the chance of reporting one or more spuriously significant results is high. For this reason, patterns of results, rather than isolated findings, are emphasized in reporting and interpreting the study results, particularly with respect to the satisfaction items. With the exception of tests of the significance of differences between dependent correlations, which were carried out using the SISA online calculator [17], all analyses were carried out using SPSS version 13.0 [18].

## Results

### Site Characteristics

Site characteristics are summarized in Table 1. Site ownership was distributed relatively evenly between individuals and organizations. Only a minority of the sites had an editorial board, and a health professional was involved in fewer than 40% of the sites. The majority of the sites were focused specifically on the topic of depression as might be expected from sites selected from a depression directory, although one-third contained more general mental health or health content. Over 40% of the sites promoted some type of product or service. Just under one-third of the sites collected personal information, and one-quarter required registration in order to obtain all of the site’s information. One-third of the sites did not publish a privacy policy. Surprisingly, over 40% failed to include a disclaimer (eg, a statement that the website was not intended as a substitute for medical advice).

### Level of Quality and Satisfaction

Overall, the mean evidence-based score was low (3.6, SD = 3.9), and the mean DISCERN ratings for both the health professional and consumer raters fell in the poor to average range (health professionals: mean = 37.8, SD = 17.0; consumers: mean =36.3, SD = 10.6). Mean satisfaction scores were low for the evidence-based raters (mean = 2.8, SD = 2.1), were average for the health professional DISCERN raters (mean = 4.3, SD = 2.6), and average for the consumer DISCERN raters (rater 1: mean = 6.1, SD = 2.3; rater 2: mean = 4.7, SD = 3.0; rater 3: mean = 4.4, SD = 3.7).

**Table 1:** Site characteristics and evidence-based quality scores

**Site Characteristic**		**Number of Sites (%)**	**Mean (SD) Evidence-Based Guideline Score(max = 20)**
**Ownership structure**	Individual	13 (54.2%)	1.5 (2.1)
	Organization*	11 (45.8%)	6.1 (4.2)
			t_14.2_** = −3.36, *P* = .005
**Editorial board**	Yes	6 (25%)	7.7 (3.2)
	No	18 (75%)	2.2 (3.1)
			t_22_ = −3.67, *P* = .001
**Scope**†	Depression specific	15 (62.5%)	2.4 (3.5)
	Broad scope	8 (33.3%)	6.2 (3.7)
			t_21_ = −2.41, *P* = .03
**Health professional involved**	Yes	9 (37.5%)	7.2 (3.0)
	No	15 (62.5%)	1.4 (2.6)
			t_22_ = −4.94, *P* < .001
**Promotion of products/services**	Yes	10 (41.7%)	4.1 (4.2)
	No	14 (58.3%)	3.2 (3.8)
			t_22_ = −0.54, *P* = .596
**Privacy policy**	Yes	9 (37.5%)	6.4 (3.6)
	No	15 (62.5%)	1.9 (3.1)
			t_22_ = −3.23, *P* = .004
**Disclaimer**	Yes	10 (58.3%)	7.0 (3.7)
	No	14 (41.7%)	1.2 (1.7)
			t_11.7_** = −4.64, *P* = .001
**Feedback mechanism**	Yes	22 (91.7%)	N/A‡
	No	2 (8.3%)	N/A‡
**Register for all information**	Yes	6 (25%)	6.6 (4.0)
	No	18 (75%)	2.6 (3.4)
			t_22_ = −2.38, *P* = .03
**Collect personal information**	Yes	7 (29.2%)	4.6 (4.1)
	No	17 (70.8%)	3.1 (3.9)
			t_22_ = −0.85, *P* = .41
**All sites**		24	3.6 (3.9)

^*^ Commercial, consumer, or other organized group

^**^ Levene's correction applied

^†^ One site not depression related

^‡^ Not analyzed due to small sample size

### Association Between Evidence-Based Quality and the Potential Indicators of Quality

#### DISCERN

There was a strong correlation between the average evidence-based score and the average DISCERN ratings for the health professionals (*r* = 0.80, *P* < .001) and a moderately high correlation for consumers (*r* = 0.62, *P* = .002). For health professionals, intercorrelations between DISCERN ratings and evidence-based scores for each of the items considered separately ranged from 0.37 (*P* = .08) for Item 5 (Is it clear when the information used or reported in the publication was produced?) to 0.88 for Item 3 (*P* < .001) (Is it relevant? eg, Does the publication address the questions readers might ask and are the treatment recommendations realistic or appropriate?). For consumers, this range was 0.18 (*P* = .40) for Item 5 to 0.68 (*P* < .001) for Item 3.

#### Google PageRank

There was a moderate correlation between the evidence-based guideline score and Google PageRank (*r* = 0.59, *P* = .002). The size of this correlation was almost the same as that between the consumer DISCERN ratings and evidence-based scores.

#### Site Characteristics

Table 1 shows the evidence-based guideline scores as a function of site characteristics. Evidence-based quality was significantly higher for organizations, sites with an editorial board, sites with broad health content, and sites involving a health professional than for their counterparts. Similarly, sites which posted a privacy policy, sites which included a disclaimer, and sites requiring registration to obtain all information were of significantly higher evidence-based quality. There was no significant difference in evidence-based guideline scores for sites that promoted products or services or that collected personal information on visitors.

### Associations Between Quality Measures and Satisfaction

Evidence-based ratings were significantly correlated with overall rater satisfaction (*r* = 0.85, *P* < .05). Sites that were judged by consumers to have useful treatment information, to describe what a consumer might wish to know about depression, to be trustworthy, and to be written by people who knew about depression showed better evidence-based quality, at least for 2 of the 3 consumers (Table 2). There were no significant differences in evidence-based scores for consumers as a function of the judged attractiveness of the site or whether they would recommend it to someone else. Sites judged by health professional raters as useful, relevant, written by a knowledgeable author, and worthy of recommendation were of higher evidence-based quality. There were no significant differences in evidence-based scores as a function of whether the site was judged by health professionals to be navigable. The pattern of findings for the health professionals who provided evidence-based ratings was similar to the pattern of findings for health professionals who conducted DISCERN ratings.

**Table 2:** Mean DISCERN scores for consumers and mean evidence-based and DISCERN scores for health professionals, as a function of individual satisfaction items

	**Consumer Raters**			**Health Professional Raters**			
				**Evidence-Based**		**DISCERN**	
**Item**	**Rater 1**	**Rater 2**	**Rater 3**	**Rater 1**	**Rater 2**	**Rater 1**	**Rater 2**
**Useful treatment**							
Yes No	6.45 (n = 10) 1.54 (n = 14) t_22_ = −3.83 *P* = .001	6.17 (n = 9) 2.14 (n = 7) t_14_ = −2.48 *P* = .03	4.45 (n = 11) 2.85 (n = 13) t_22_ = −1.00 *P* = .32	8.93 (n = 7) 1.38 (n = 17) t_22_ = −9.46 *P* < .001	8.93 (n = 7) 1.38 (n = 17) t_22_ = −9.46 *P* < .001	7.50 (n = 9) 1.23 (n = 15) t_10.6_* = −5.22 *P* < .001	5.82 (n = 11) 1.09 (n = 11) t_13.97_* =−3.75 *P* = .002
**Useful overall**							
Yes No	4.63 (n = 16) 1.50 (n = 8) t_22_ =−1.96 *P* = .06	5.23 (n = 11) 2.19 (n = 13) t_22_= −2.02 *P* = .06	4.04 (n = 12) 3.13 (n = 12) t_22_ = −.57 *P* = .58	7.33 (n = 9) 1.33 (n = 15) t_9.93_* = −4.57 *P* = .001	8.93 (n = 7) 1.38 (n = 17) t_22_ = −9.46 *P* < .001	6.73 (n = 11) .92 (n = 13) t_13.7_* = −5.11 *P* < .001	6.89 (n = 9) 1.08 (n = 13) t_10.8_* = −4.92 *P* < .001
**Relevant**							
Yes No	6.94 (n = 8) 1.90 (n = 16) t_22_ =−3.70 *P* = .001	6.71 (n = 7) 2.29 (n = 17) t_22_ = −2.89 *P* = .008	3.88 (n = 13) 3.22 (n = 11) t_15.71_* = −.39 *P* = .70	10.0 (n = 1) 3.30 (n = 23) –	9.90 (n = 5) 1.92 (n = 19) –	8.75 (n = 6) 1.86 (n = 18) t_22_ = −5.83 *P* < .001	8.19 (n = 8) 1.28 (n = 16) t_22_ =−7.58 *P* < .001
**Trustworthy**							
Yes No	5.40 (n = 15) .56 (n = 9) t_17.14_* = −4.57 *P* < .001	6.71 (n = 7) 2.29 (n = 12) t_17_= −3.14 *P* = .006	4.77 (n = 13) 2.18 (n = 11) t_22_ = −1.68 *P* = .11	7.20 (n = 5) 2.63 (n = 19)	9.25 (n = 4) 2.58 (n = 19)	6.22 (n = 9) 2.00 (n = 15) t_22_ = −2.96 *P* = .007	5.07 (n = 14) 1.88 (n = 8) t_20_ =−1.96 *P* = .07
**Knowledgeable**							
Yes No	4.58 (n = 18) .58 (n = 6) t_21.97_* = −3.76 *P* = .001	5.81 (n = 11) 1.83 (n = 12) t_21_ = −2.78 *P* = .01	4.93 (n = 15) 1.50 (n = 8) t_21_ = −2.16 *P* = .04	8.57 (n = 7) 1.53 (n = 17) t_22_ = −7.15 *P* < .001	8.57 (n = 7) 1.53 (n = 17) t_22_ = −7.15 *P* < .001	5.82 (n = 14) .45 (n = 10) t_15.4_* = −5.23 *P* < .001	5.34 (n = 16) .08 (n = 6) t_15.2_* = −5.70 *P* < .001
**Understandable**							
Yes No	3.58 (n = 24) – (n = 0) –	3.74 (n = 23) 0 (n = 1) –	4.55 (n = 10) 2.89 (n = 14) t_22_ = −1.024 *P* = .32	2.27 (n = 13) 5.14 (n = 11) t_14.85_* = 1.80 *P* = .09	4.08 (n = 19) 1.70 (n = 5) –	3.90 (n = 22) 0 (n = 2) –	4.38 (n = 17) 1.64 (n = 7) t_20.2_* = −2.07 *P* = .05
**Navigable**							
Yes No	3.3 (n = 23) 10 (n = 1) –	3.95 (n = 21) 1 (n = 3) –	3.79 (n = 12) 3.38 (n = 12) t_22_ = −.26 *P* = .80	3.2 (n = 16) 4.3 (n = 8) t_22_ = .64 *P* = .53	3.84 (n = 16) 3.50 (n = 7) t_21_ = −.19 *P* = .85	3.08 (n = 18) 5.08 (n = 6) t_22_ = 1.09 *P* = .29	2.98 (n = 20) 6.63 (n = 4) t_22_ = 1.78 *P* = .09
**Attractive**							
Yes No	3.74 (n = 23) 0 (n = 1) –	3.92 (n = 13) 3.18 (n = 11) t_22_ = −.46 *P* = .65	3.93 (n = 7) 3.44 (n = 17) t_22_ = −.27 *P* = .79	6.80 (n = 5) 2.81 (n = 18) –	1.83 (n = 3) 3.92 (n = 19) –	6.42 (n = 7) 2.41 (n = 17) t_22_ = −2.55 *P* = .02	4.25 (n = 4) 3.45 (n = 20) –
**Recommended**							
Yes No	5.39 (n = 9) 2.5 (n = 15) t_22_ =−1.84 *P* = .08	4.65 (n = 10) 2.19 (n = 13) t_21_ =−1.66 *P* = .11	4.04 (n = 12) 3.13 (n = 12) t_22_ = −.57 *P* = .58	10.83 (n = 3) 2.60 (n = 20) –	4.25 (n = 2) 3.39 (n = 19)	8.75 (n = 6) 1.86 (n = 18) t_22_ = −5.83 *P* < .001	7.36 (n = 7) 1.83 (n = 15) t_20_ = −4.00 *P* = .001

^*^ Levene's correction applied

Consumer DISCERN ratings were strongly correlated with satisfaction ratings (rater 1: *r* = 0.74, *P* < .001; rater 2: *r* = 0.85, *P* < .001) as were expert DISCERN ratings (*r* = 0.95, *P* < .001). By contrast, PageRank was correlated with consumer satisfaction for one rater only (rater 1: *r* = 0.45, *P* = .03; rater 2: *r* = 0.35, *P* > .05; rater 3: *r* = 0.21, *P* > .05) and was only moderately correlated with expert satisfaction ratings (*r* = 0.50, *P* = .01). This difference in correlation for the DISCERN and PageRank conditions was significant for two of the consumers and also for the health professionals (consumer rater 1: difference in *r* = 0.29, 95% CI = −0.02 to 0.60); rater 2: difference in *r* = 0.50, 95% CI = 0.18 to 0.82; rater 3: difference in *r* = 0.64, 95% CI = 0.26 to 1.02; health professionals: difference in *r* = 0.45, 95% CI = 0.23 to 0.67)

## Discussion

This study provides the first published demonstration that DISCERN is an indicator of evidence-based website quality when used by consumers. It also confirms our previous finding [4] that DISCERN is an indicator of evidence-based quality when used by health professionals.

The finding that DISCERN may be a valid means for consumers to identify websites of high quality and satisfaction has practical implications for consumers. It is unlikely that individual consumers will invest the time required to use DISCERN solely for their own purposes. However, used with caution and an understanding that it is not a perfect predictor of evidence-based quality, DISCERN may be relevant to consumer organizations interested in assembling lists of links to high quality websites for their membership or for visitors to their website. Moreover, the finding that DISCERN may be useful for consumers raises the possibility that DISCERN might also be validly applied by other nontechnical experts, an observation of potential relevance to any organization or Web constructor interested in inexpensively assembling quality portals.

Interestingly, in the case of consumers, Google PageRank is as strong an indicator of evidence-based quality as DISCERN. Thus, this measure may be a simple and practical means by which individual consumers can evaluate, albeit imperfectly, the likely quality of mental health sites. Apart from the time required to download the Google toolbar in the first instance, its use requires minimal expertise and time. In addition, PageRank is likely to be convenient for users seeking health information on the Web since they typically do so by means of a search rather than via directories or portals [19,20]. Since the Google PageRank was correlated less highly with satisfaction than was DISCERN, the latter may be the preferred rating tool for organized groups for whom the overhead in learning to use DISCERN can be justified. However, even in this circumstance, it is possible that Google PageRank could be used as a screening device to eliminate likely sites of low quality and the more time consuming DISCERN instrument then applied to the remaining sites. Alternatively, the reduction in sites may render the task of assessment by a content expert feasible.

It is encouraging that sites regarded by consumers as more useful, trustworthy, and relevant are, on average, sites of higher evidence-based quality. This suggests that consumers’ own judgment of and satisfaction with website content may be a useful indicator of appropriate sites. Finally, consumers might be guided by the finding from this and two of our previous studies [4,21] that sites produced by organizations and sites that have an editorial board are of above-average quality. In addition, consumers may be able to place more reliance on sites that pay attention to factors such as a privacy policy, a disclaimer, feedback mechanisms, and on sites that involve health professionals. By contrast, stylistic attributes (eg, judged attractiveness) do not appear to be a useful basis for identifying higher quality sites.

### Limitations

This study suffers from several limitations. First, considerable caution is needed in applying the results given that the correlations between the evidence-based scores and DISCERN and Google PageRank were of the order of 0.6 for the consumers. Although considered a strong relationship in the behavioral sciences [22], correlations of this magnitude will result in misclassifications, including false positives and false negatives. Second, the number of consumers employed in the study was small. Third, the study was confined to the field of depression. Fourth, a study of the psychometric properties of the satisfaction measure has not been undertaken. It is therefore difficult to determine if the lower agreement between satisfaction and DISCERN among consumers reflects inadequate reliability of the measure for consumers or a greater variability among consumers than health professionals as to what constitutes satisfaction. In addition, consumer scores on this measure may have been influenced by their concurrent use of DISCERN. Similarly, satisfaction ratings provided by the evidence-based health professional raters may have been influenced by their prior coding of site characteristics and ratings of evidence-based quality. It would therefore be appropriate to repeat the study with a larger number of consumers and health professionals, to employ a design in which the ratings on different instruments, such as DISCERN and satisfaction, were each provided by different consumers and health professionals using a validated, reliable measure of satisfaction, and to determine if the findings are robust across a range of mental health and other health domains. In addition, although a number of site characteristics were associated with better evidence-based quality, the website sample size was insufficient to conduct analyses to identify the independent effects of these characteristics on quality. It is possible, for example, that organizations are more likely to both produce high-quality sites and incorporate a privacy policy, disclaimer, and feedback mechanism. Finally, none of the raters—professional or consumer—were experienced in the use of the DISCERN instrument. The findings may therefore underestimate the usefulness of DISCERN as an indicator of quality when used by an experienced rater.

### Conclusions

These results represent a first step toward identifying tools that consumers who are not content experts can use as valid indicators of the evidence-based quality of websites. Further research is required to explore the utility of DISCERN and Google PageRank. In particular, it is important to determine optimal cutoff points for identifying higher quality sites and to explore the sensitivity and specificity of the measures. It is also of interest to document the relative utility of DISCERN for nontechnical raters of differing educational backgrounds, experience with the instrument, and Web experience. Finally, given that not one but many indicators may be useful in identifying high-quality sites, there may be value in identifying optimal combinations of multiple indicators of quality. There is also much to be gained by further identifying automatic indicators of the type that could be factored into the relevance algorithms of a specialized focused search engine.
